# Association of Lipid Profile and Suicide Attempts in a Large Sample of First Episode Drug-Naive Patients With Major Depressive Disorder

**DOI:** 10.3389/fpsyt.2020.543632

**Published:** 2020-09-30

**Authors:** Yue-Jiao Ma, Yong-Jie Zhou, Dong-Fang Wang, Yi Li, Dong-Mei Wang, Tie-Qiao Liu, Xiang-Yang Zhang

**Affiliations:** ^1^ Department of Psychiatry, The Second Xiangya Hospital, Central South University, The China National Clinical Research Center for Mental Health Disorders, Chinese National Technology Institute of Psychiatry, Key Laboratory of Psychiatry and Mental Health of Hunan Province, Changsha, China; ^2^ Department of Addictive Behavior and Addiction Medicine, Medical Faculty Mannheim, Central Institute of Mental Health, Heidelberg University, Mannheim, Germany; ^3^ Research Center for Psychological and Health Sciences, China University of Geosciences, Wuhan, China; ^4^ Affiliated Wuhan Mental Health Center, Tongji Medical College of Huazhong University of Science & Technology, Wuhan, China; ^5^ Department of Psychiatry and Psychotherapy, Technical University of Munich, Munich, Germany; ^6^ CAS Key Laboratory of Mental Health, Institute of Psychology, Chinese Academy of Sciences, Beijing, China; ^7^ Department of Psychology, University of Chinese Academy of Sciences, Beijing, China

**Keywords:** major depressive disorder, lipid, suicide attempt, clinical symptom, first episode

## Abstract

Several studies have reported a link between lipid disorders and suicidality. However, few studies have investigated the relationship between suicidal behavior and blood lipid profiles in patients with first-episode and drug-naive (FEDN) major depressive disorder (MDD). The main purpose of this study was to examine the relationship between plasma lipid profiles and suicide attempts in a large sample of FEDN MDD patients in the Chinese Han population, which has not been reported. A total of 1,718 MDD outpatients were recruited. Their clinical and demographic data as well as plasma lipid parameters were collected. We obtained suicide attempt data through interviews with patients and their family members. We rated the Hamilton Depression Scale (HAMD) and Hamilton Anxiety Scale (HAMA) for all patients. The suicide attempt rate of MDD patients was 20.14%, of which 13.68% in the last month and 6.46% in the past. Further, compared with non-attempters, suicide attempters had significantly higher total levels of cholesterol (TC) and low-density lipoprotein cholesterol (LDL-c), higher HAMA and HAMD scores, but significantly lower high-density lipoprotein cholesterol (HDL-c) levels. Logistic regression analysis showed that suicide attempts were correlated with higher TC, lower HDL-c, and higher HAMA and HAMD scores with the adjusted odds ratio (OR) of 1.35, 0.52,1.28, and 1.08, respectively (all p < 0.05). Our findings suggest that FEDN patients with MDD have a high rate of attempted suicide. In the early stage of MDD patients, certain blood lipid parameters and more severe symptoms of anxiety and depression are correlated with suicide attempts. However, due to the cross-sectional design of this study, it is impossible to draw a causal relationship between lipid profiles and suicide attempts. Moreover, an inverse correlation can also be considered, that is, high cholesterol may be the consequence of suicide attempts and depression.

## Introduction

In China, the suicide attempt rate of patients with major depressive disorder (MDD) is reported to be 7.3 to 48.4% (1), and in other countries, it is 16.9 to 33.7% (2–4). Moreover, more than 90% of people who die by suicide suffer from one or more mental illnesses, especially MDD, accounting for 59–87% of all suicides (1, 5). A systematic review suggests that many confounding factors may contribute to the risk of suicide in MDD patients, such as gender, family history of mental illness, more severe depression and comorbidities (6). In the past decades, many researchers have been trying to identify biomarkers that may be related to suicidal behavior. The role of lipid disorders in suicide has always been one of the researchers’ focuses (7, 8). For example, a previous study found that the number of deaths among subjects treated with cholesterol-lowering drugs increased significantly, including suicide deaths (9). Subsequently, many studies have investigated the relationship between total cholesterol (TC) levels and suicide, but the results are conflicting (10–14). The association between low TC levels and suicide has been found in MDD (15–18), schizophrenia (19), personality disorder (20), anorexia nervosa (21), panic disorder (22), and bipolar disorder (23), or even in the general population (24), suggesting that low lipid metabolism, especially TC, may be a potential biomarker of suicidal behavior.

A large number of epidemiological and clinical studies have shown that MDD subjects with lower TC levels have increased suicide attempts (15–18, 25). For example, Papadopoulou et al. reported that psychiatric patients with a history of suicide attempts (including MDD) have lower TC levels. During their follow-up, the TC levels remained low (18). Moreover, in depression patients suffering from suicidal behavior, LDL-c, TC levels (26) and triglycerides (TG) levels (27, 28) were significantly lower. However, it has been reported that patients with depression who attempt suicide have higher HDL-c levels (27, 29). Further, some studies have shown that there is no relationship between the blood lipid levels and suicidal behavior in patients with depression (30–32), or even the opposite results in community populations (13, 33) and depression patients (15, 34). For example, Brunner et al demonstrated that in the past 12 months, there was a positive correlation between TC or TG and attempted suicide in patients with depression (34). A recent longitudinal study has found that both low and high TC levels can predict the incidence of suicidal ideation in the elderly, regardless of life stress, social support, alcohol consumption, depressive symptoms, and disability (35). Taken together, the relationship between suicidal behavior and blood lipid levels in MDD patients is still inconsistent.

Most of previous studies have had fewer clinical samples. Moreover, most of the subjects in previous studies were hospitalized or taking medication, which may affect diet and exercise habits, as well as subsequent lipid levels (15, 36, 37). Interestingly, it was reported that patients with first episode mental illness had a higher risk of suicide (38). Moreover, 60% of depressed patients completed suicide at their first episode (39). However, to our best knowledge, there is no study investigating the relationship between suicide attempt and lipid levels in first episode-depressed patients in a large sample. Moreover, it is unclear whether lipid level is either a state or a trait marker of suicide attempt.

In this study, we recruited a large sample of first-episode drug-naive (FEDN) MDD patients (n = 1,718) in a Chinese Han population. It is worth noting that the study of patients with first-episode MDD is helpful to understand the relationship between suicide attempts and lipid metabolism in MDD, which may minimize confounders, including drug treatment, course of disease, and related mental illness and medical complications. Therefore, the purposes of this study were (1) to investigate the prevalence and clinical features of attempted suicide in FEDN MDD patients, and (2) to identify contributors that are significantly associated with attempted suicide in these MDD patients.

## Methods

### Subjects

This was a cross-sectional naturalistic study conducted in a psychiatric outpatient department of a general hospital in Taiyuan, Shanxi Province, China. 1,718 patients were recruited and met the following inclusion criteria: 1) Han nationality; 2) aged between 18 and 60 years; 3) meeting MDD diagnosis in accordance with the Diagnostic and Statistical Manual of Mental Disorders (DSM) IV-TR; 4) at the first onset of MDD; 5) before entering the study, did not receive any drugs, including antidepressants, anti-anxiety drugs, antipsychotics, and other drugs that affect lipid levels, such as lipid-lowering drugs; 6) except for smoking, no alcohol or drug abuse disorders; 7) no pregnancy or lactation; 8) no major medical abnormalities, including central nervous system diseases and acute, unstable or life-threatening medical diseases such as cancer and infections.

The study was approved by the Institutional Review Board (IRB) of the First Clinical Medical College, Shanxi Medical University. All subjects signed an informed consent form to participate in this study.

### Socio-Demographic Characteristics and Clinical Measures

Demographic and clinical data were collected by well-trained researchers. Each patient completed a detailed questionnaire, including general information and sociodemographic characteristics. We also collected other information from available medical records.

Suicidal behavior includes attempted suicide or suicide. Complete suicide (synonymous with suicide) is the act of deliberately causing one’s own death. The definition of attempted suicide is a self-destructive behavior aimed at ending a person’s life without causing death. In this study, the main outcome measure was attempted suicide. All participants were asked “In your entire lifetime did you ever attempt suicide?” If they gave an affirmative answer, they were coded as suicide attempter. They were then asked about their previous suicide attempts, including the following details: the number of suicide attempts, the exact date and method of each suicide attempt. If the answer was ambiguous, the researcher made additional visits with the patients’ family members or clinical team for confirmation. The suicide attempters were further divided into two subgroup s: those who attempted suicide in the last month (recent suicide group), and those who attempted suicide in the past except for the last month (past suicide group).

The Hamilton Depression Scale (HAMD, 17 items) and Hamilton Anxiety Scale (HAMA, 14 items) were assessed by trained psychiatrists. In order to ensure the consistency and reliability of the scores throughout the study, two psychiatrists received training on the use of HAMD and HAMA before the study. After training, repeated evaluations showed that the inter-rater correlation coefficient of HAMD and HAMA total scores was greater than 0.8. Two psychiatrist assessors were blind to the patient’s clinical condition.

### Blood Sample

On the day when the clinical rating scale was measured, blood samples were collected between 6 am and 7 am after an overnight fast. All samples were sent to the laboratory center of the hospital immediately and measurements were taken before 11 am in the morning of the same day. The plasma TC, TG, HDL-c, and LDL-c levels were measured on the BECKMAN AU5800 system (Beckman Coulter, Brea, California, USA).

### Statistical Analysis

The prevalence of attempted suicide was expressed as a percentage. The demographic and clinical variables of the recent suicide group, past suicide group and non-suicide group were compared by using the analysis of variance (ANOVA) for continuous variables and the chi-square test for categorical variables. Since there was no significant difference in lipid distribution between the recent suicide group and the past suicide group, we combined these two subgroups into one suicide attempt group. Then, we compared the demographic and clinical variables between suicide attempters and non-attempters by performing t-tests for continuous variables and chi-square tests for categorical variables. Whereas there was a significance in ANOVA test, analysis of covariance (ANCOVA) was then conducted to control for the influence of confounding factors. Finally, after controlling for the effects of age, gender, BMI, age of onset, duration of illness, severity of depression symptoms (HAMD) and severity of anxiety symptoms (HAMA), binary logistic regression analysis (enter) was performed to assess the factors associated with suicide attempts. Statistical analyses were performed using SPSS 22.0 for Windows. All statistical tests were two-tailed, and the significance level was set at ≤0.05.

## Results

A total of 1,718 patients included 588 men and 1,130 women. Among them, 346 (20.14%) had a history of attempted suicide, of whom 235 (13.68%) had attempted suicide in last month and 111 (6.46%) had attempted attempt in the past except for the last month. Their average age was 34.87 ± 12.43 years and the average course of disease was 6.31 ± 4.73 months, with a range of 5 to 28 months.

**Table 1** shows the socio-demographic and clinical data of the recent suicide group, past suicide group, and non-suicide group. There were significant differences in sex (χ^2^ = 7.92, p = 0.02), duration of illness (F = 6.87, p < 0.001) and BMI (F = 12.30, p < 0.001) between the three groups. Compared with the past suicide group, the recent suicide group had more women (p = 0.02), shorter duration of illness (p = 0.04), and higher BMI level (p < 0.001).

**Table 1:** Comparison of characteristics between recent and past suicide attempters and non-attempters (n = 1,718).

	Recent suicide attempters^1^	Past suicide attempters^2^	Never suicide attempters^3^ (n = 1372)	χ2/F	*P*
	(n = 235)	(n = 111)			
**Socio-demographic and clinical**					
**Characteristics (Mean ± SD)**					
Age (years)	36.48(12.57)	35.36(11.90)	34.55(12.43)	2.51	0.08
Female (%)	170(72.34%)	64(57.66)	896(65.30%)	7.92	0.02^a^
Unmarried (%)	60(25.53%)	35(31.53%)	407(29.66%)	1.98	0.37
Education (years)	12.61(3.19)	12.43(2.84)	12.76(2.89)	0.79	0.45
Age of onset (years)	36.28(12.49)	35.06(11.79)	34.35(12.31)	2.51	0.08
Duration of illness (month)	6.53(4.84)	7.83(4.88)	6.15 (4.68)	6.87	0.00^b^
BMI	24.67(1.73)	23.58(3.13)	24.38(1.81)	12.30	0.00^c^
**Scale assessment (Mean ± SD)**					
HAMD	32.09(2.75)	32.55(3.14)	29.81(2.75)	107.17	0.00^d^
HAMA	24.22(3.55)	22.25(3.15)	20.09(3.08)	184.67	0.00^e^
**Biological indicators (Mean ± SD)**					
Total Cholesterol (mmol/L)	5.82(1.12)	5.67(1.08)	5.12(1.07)	52.61	0.00^f^
Triglycerides (mmol/L)	2.30(1.03)	2.29(0.98)	2,14(0.98)	3.59	0.00^g^
HDL cholesterol (mmol/L)	1.14 (0.28)	1.14 (0.30)	1.24(0.28)	18.32	0.00^h^
LDL cholesterol (mmol/L)	3.20 (0.92)	3.23 (0.88)	2.92(0.84)	15.25	0.00^i^

BMI, body mass index; HAMD, Hamilton Depression Scale; HAMA, Hamilton Anxiety Scale; HDL, high density lipoprotein; LDL, low density lipoprotein.

All post-hoc analyses were done using Tukey;

a ^1^vs.^2^ p = 0.02,^1^vs.^3^ p = 0.09, ^2^vs.^3^ p = 0.23;

b ^1^vs.^2^ p = 0.04,^1^vs.^3^ p = 0.48, ^2^vs.^3^ p < 0.001;

c ^1^vs.^2^ p < 0.001,^1^vs.^3^ p = 0.08, ^2^vs.^3^ p < 0.001;

d ^1^vs.^2^ p = 0.33,^1^vs.^3^ p < 0.001, ^2^vs.^3^ p < 0.001;

e all post-hoc test p < 0.001;

f ^1^vs.^2^ p = 0.46,^1^vs.^3^ p < 0.001,^2^vs.^3^ p < 0.001;

g ^1^vs.^2^ p = 1.00,^1^vs.^3^ p = 0.05,^2^vs.^3^ p = 0.27;

h ^1^vs.^2^ p = 1.00,^1^vs.^3^ p < 0.001,^2^vs.^3^ p < 0.001;

i ^1^vs.^2^ p = 0.95, ^1^vs.^3^ p < 0.001,^2^vs.^3^ p < 0.001.

There were significant differences between the three groups, including TC (F = 52.61, p < 0.001), TG (F = 3.59, p < 0.001), HDL (F = 18.32, p < 0.001), LDL (F = 15.25, p < 0.001), HAMD (F = 107.17, p < 0.001), and HAMA (F = 184.67, p < 0.001). *Post hoc* analysis showed that TC and LDL levels, HAMD and HAMA scores of the recent suicide group and past suicide group were significantly higher than those of the non-suicide group (all p < 0.001). Moreover, HDL levels of the recent and past suicide groups were significantly lower than that of the non-suicide group (p < 0.001). However, there were no significant differences in lipid parameters and HAMD and HAMA scores between the recent and past suicide groups (all p > 0.001).

Since there were no significant differences in lipid parameters between the recent and past suicide groups, we combined them into one suicide group to compare the lipid profiles between suicide and non-suicide groups. **Table 2** shows the socio-demographic and clinical data of these two groups. Suicide attempters were significantly older (t = −2.10; p = 0.036), had a later age of onset (t = 0.66; p = 0.04), longer duration of illness (t = −2.83; p = 0.01), higher levels of TC (t = 10.19; p < 0.001), LDL-c (t = −5.51; p < 0.001) and TG (t = −2.68;p = 0.008), higher HAMA (t = −16.83; p < 0.001), and HAMD scores (t = −14.57; p < 0.001) but lower HDL-c levels (t = 6.05; p < 0.001) compared with non-attempters. After controlling for sex, age, and BMI, further ANCOVA showed that the following parameters between the two groups still remained significant: age of onset (F = 1.91; p < 0.001), TC (F = 1.47; p < 0.001), LDL-c (F = 1.51; p < 0.001) and TG (F = 1.27; p = 0.001), HDL-c (F = 1.45; p = 0.001), HAMA (F = 18.50; p < 0.001), and HAMD scores (F = 14.45; p < 0.001). In addition, there was no significant correlation between the number of suicide attempts and any lipid profiles (all p > 0.05).

**Table 2:** Characteristics of first episode drug naïve depressed patients with/without suicide attempts (n = 1718).

	All patients (n = 1,718)	Suicide attempt	Non-suicide attempt	t/χ2	P
		(n = 346)	(n = 1,372)		
**Socio-demographic and Clinical Characteristics (Mean ± SD)**					
Age (years)	34.87(12.43)	36.12 (12.35)	34.55 (12.43)	−2.10	0.036
Female (%)	1130(65.77%)	234 (67.60%)	896 (65.30%)	0.66	0.42
Unmarried (%)	502(29.22%)	95 (27.50%)	407(29.70%)	0.65	0.42
Education (years)	12.72(2.93)	12.55(3.08)	12.76(2.89)	1.14	0.25
Age of onset (years)	34.66(12.31)	35.89 (12.26)	34.35 (12.31)	0.66	0.04
Duration of illness (month)	6.31(4.73)	6.95 (4.89)	6.15 (4.68)	−2.83	0.01
BMI	24.37(1.93)	24.32(2.33)	24.38(1.81)	0.43	0.67
**Scale assessment (Mean ± SD)**					
HAMD	30.30(2.94)	32.24(2.89)	29.81(2.75)	−14.57	0.001
HAMA	20.80(3.47)	23.59(3.54)	20.09(3.08)	−16.83	0.001
**Biological indicators (Mean ± SD)**					
Triglycerides (mmol/L)	2.17(0.99)	2.29(1.02)	2.14(0.98)	−2.68	0.008
HDL cholesterol (mmol/L)	1.22(0.29)	1.14(0.29)	1.24(0.28)	6.05	0.001
LDL cholesterol (mmol/L)	2.98(0.86)	3.21(0.91)	2.93(0.84)	−5.51	0.001
Total Cholesterol (mmol/L)	5.25(1.11)	5.77(1.11)	5.12(1.07)	10.19	0.001

BMI, body mass index; HAMD, Hamilton Depression Scale; HAMA, Hamilton Anxiety Scale; HDL, high density lipoprotein; LDL, low density lipoprotein.

Further binary logistic regression analysis showed that higher TC, lower HDL-c, and higher HAMA and HAMD scores were significantly associated with attempted suicide (**Table 3**), with the adjusted odds ratio (OR) of 1.28 (standardized *β* = 0.24; 95% CI: 0.12–1.35; df = 1, p < 0.01) for HAMA, 1.08 (standardized *β* = 0.08; 95%CI:0.11–1.16; df = 1, p = 0.01) for HAMD and1.35 (standardized *β* = 0.30; 95%CI: 0.11–1.59; df = 1, p < 0.01) for TC, and 0.52 (standardized *β* = −0.65; 95% CI: 0.33–0.83; df = 1, p < 0.01) for HDL-c.

**Table 3:** Predictors of suicide attempts within a binary logical regression model.

	Coefficients				95.0% Confidence Interval	
	*β*	Odds Ratio	*z* value	*p* value	Lower	Upper
Age	−0.37	0.69	−1.35	0.18	0.40	1.19
Gender	0.03	1.03	0.18	0.86	0.77	1.37
BMI	−0.06	0.94	−1.35	0.18	0.88	1.01
Age of onset	0.37	1.45	1.35	0.18	0.84	2.48
Duration of illness	0.05	1.05	1.72	0.09	0.99	1.11
HAMD	0.08	1.08	2.44	0.01	0.11	1.16
HAMA	0.24	1.28	9.72	<0.01	0.12	1.35
TC	0.30	1.35	3.54	<0.01	0.11	1.59
HDL-c	−0.65	0.52	−2.73	<0.01	0.33	0.83
TG	−0.07	0.93	−0.98	0.33	0.80	1.07
LDL-c	−0.11	0.89	−1.23	0.22	0.74	1.07

HAMD, Hamilton Depression Scale; HAMA, Hamilton Anxiety Scale; TC, Total Cholesterol; HDL, high density lipoprotein; TG, Triglycerides; LDL, low density lipoprotein.

In addition, we further conducted statistical analysis and provided results based on clinical reference values. The normal range of each index is as follow: 2.83–5.17 mmol/L for TC, ≤1.7 mmol/L for TG, ≥1.04 mmol/L for HDL-C, ≤3.12 mmol/L for LDL-C. Then we classified normal TC and decreased TC into one group for analysis because there were very few people (n = 11) with TC lower than 2.83 mmol/L. The chi-square test was used to compare the metabolic indicators between attempted suicide and non-suicide participants. Compared with those without attempted suicide, those who attempted suicide had significantly higher levels of TC and LDL-c but lower HDL-C levels (all p < 0.05). Binary logistic regression analysis indicated that in MDD patients, suicide attempts were associated with HDL-c, with an OR of 0.73 (95% CI: 0.54–0.97; df = 1, p < 0.05).

## Discussion

To our best knowledge, this is the first study to investigate the association between lipid profiles and attempted suicide in such a large FEDN MDD sample in China. Our study showed that the prevalence of suicide attempts in this large group of MDD patients was 20.14%. We did not find significant difference in lipid profiles between recent and past suicide attempts. However, the lipid profiles of the two suicide groups were significantly different from those of the non-suicide group. Further analysis found that adjusting for gender, age and BMI, attempted suicide group had significant differences in demographic, clinical, and biochemical variables from non-suicide group. Further logistic regression analysis showed that higher TC levels, lower HDL-c levels, more severe anxiety and depressive symptoms were associated with suicide attempts, suggesting that these parameters may be important risk factors associated with suicide attempts.

Suicide is the most devastating result for patients with depression, with about one death in every 25 suicide attempts (40). In our study, the prevalence of suicide attempts (20.14%) was similar to that reported in other Asian countries, such as 16.9% in upper northern Thailand (4), and 19.8% in Korea (3). A recent epidemiological survey reported that the prevalence of suicide attempts was 1.3% in rural samples and 0.9% in urban samples in Beijing area (41). Therefore, our current findings indicate that the prevalence of suicide attempt in our MDD outpatients is nearly 20 times that in the general population, suggesting a dramatic increase in suicide attempt rate in first-episode MDD patients in the Chinese population. However, it is worth mentioning that most reports of suicide attempt rates in MDD patients in Western countries range from 27.5% in Europe to 36.3% in the United States (1), which is much higher than about 20% in Asian countries. Possible reasons for the different rates of suicide attempts among different ethnic groups include health resources, cultural traditions, political environment, access to psychiatric care, economic and sociocultural factors in different regions or countries (1, 42), as well as ethnic background, which is due to interethnic differences in the genotype frequency of suicide-related gene polymorphisms between Chinese and Caucasians (42).

One of the important findings in our current study is that high TC levels were associated with suicide attempts, which is consistent with several previous studies. For example, a case–control study compared psychiatric patients who attempted suicide (193 subjects) with those who did not attempt suicide (1,091 subjects) and found that high TC levels were associated with an increased risk of suicide in American men (43). A prospective follow-up study also showed that high cholesterol was associated with an elevated risk of suicide attempts in patients with depression (15). Also, similar results were found in population-based studies. For example, a previous study reported a positive association between TC levels and long-term suicide risk in a large sample of 7,309 middle-aged Japanese-American men (33). Tanskanen et al. reported that in a large general population, the adjusted relative risk of violent suicide in the highest TC group was twice that in the lowest TC group (13). Similarly, in a population-based study, Brunner et al reported higher cholesterol levels in suicide attempters than non-attempters (34). However, the reasons behind this association are still unclear. Some researchers speculate that patients with higher TC may have maladaptive nutritional behaviors, such as overeating, which may be associated to suicide attempts and suicidal ideation (34). Another possible reason is that elevated TC may be related to stroke, which is known to increase the risk of suicide (44). However, many previous studies have reported a negative or no association between TC and attempted suicide (14, 27, 45–47). Researchers have explored the possible mechanisms of this negative correlation. The hypothesis of cholesterol–serotonin–impulsivity has been proposed that low concentrations of cholesterol may reduce the exposure of serotonin receptors on the surface of brain cell membranes, and then reduce the function of serotonin receptors, thereby leading to impulsive or aggressive behavior in susceptible individuals (7, 10, 12, 48). However, studies have shown that the transmission of serotonin partly depends on brain cholesterol, but brain cholesterol is relatively isolated from changes in circulating cholesterol (19).

In our and previous studies, the possible reasons for the difference in the relationship between TC and the attempted suicide may be due to the clinical condition of the recruited samples. For example, the sample in our study was the first episode drug-naive MDD patients, while the samples in the previous studies were hospitalized MDD patients. The blood lipid levels of these hospitalized patients may be affected by drug treatment, including antidepressants (36) and second-generation antipsychotics (49). In addition, blood lipid levels may be affected by hospitalization, dietary intake, and weight loss after long-term depression (24). Other possible reasons include that the subjects in the previous study not only suffered from depression, but also suffered from other mental disorders, such as accompanied by adjustment disorder (45) and psychotic symptoms (47), which are clinical confounders for suicide attempts. Interestingly, some researchers even suggest that changes in lipid metabolism in both directions may lead to changes in serotonergic function. For example, Kim et al reported that both higher and lower TC levels at baseline predicted the rate of suicidal ideation in the elderly. They stated that decreased serotonin activity was associated with lower TC levels, and that an atherogenic lipid profile was related to higher TC levels, and both may be associated with suicidality in later life (35). Taken together, the exact relationship between TC levels and suicide attempts remains unclear. In this study, the cross-sectional design, without long-term follow-up cholesterol data, limited the interpretation of the causal relationship between blood lipid levels and suicidal behavior, that is, the results of this cross-sectional study cannot differentiate whether high cholesterol may be a consequence of suicide attempts or the underlying pathological mechanisms that may result in suicide attempts. Although there is a positive correlation between high lipid levels and suicide attempts, the current results cannot lead to a causal relationship. Therefore, further prospective studies are needed to explore the correlation between high cholesterol and suicide attempts, which may also be confirmed by longitudinal data.

Generally, proatherogenic lipids (TC, LDL, and TG) and anti-atherogenic lipid (HDL) may have different roles in suicide. In this study, we also found a negative correlation between HDL-c and suicide attempts. However, previous studies are inconsistent, suggesting that higher or lower HDL-c levels were associated with suicide attempts in patients with depression (27, 29). The two most important findings of our study were the association between suicide attempts and high TC/low HDL-c levels. These findings are in line with the hypothesis that abnormal lipid profiles may be a sign of other metabolic abnormalities associated with increased risk of suicidal behavior. For example, omega-3 polyunsaturated fatty acids (PUFAs) may be a key mediator between lipids and suicide (34). There was an inverse correlation between PUFAs and the ratio of serum TC/HDL-c. As a result, subjects with high TC and low HDL-c have lower levels of PUFAs, and it has been reported that reduced PUFAs may be a strong risk factor for suicide and depression (50, 51), suggesting that lipid changes may be a surrogate marker.

It is worth mentioning that previous studies have found an association between the lipid profiles and either recent or lifetime suicide attempts in patients with depression (25, 27). However, whether lipid parameters are the state or trait markers for suicide attempts remain uncertain. In our current study, there was no significant difference in lipid profiles between recent suicide and past suicide attempters, suggesting that lipid parameters may be trait markers of attempted suicide. However, all patients in this study had their first onset, with an average ill duration of 7.8 months, and even those patients in the past suicide group attempted suicide in the past several months. Thus, the interval period of suicide attempts between recent and past suicide groups may not be sufficient to show differences in lipid profile. Therefore, whether the lipid profile is a state or a trait marker of the suicide attempt deserves further investigation.

Further, we found that the severity of depression was an important factor of suicide attempts, which is consistent with most previous studies (52). However, other studies have reported no correlation between suicide attempt and the severity of depression (2). The possible reason for this discrepancy may be due to the different time of assessing the severity of depression, rather than the time of attempted suicide (2). This may also explain why the Exp (β) value was only 1.08 in our current study.

Another finding of this study was that there was a significant association between anxiety and an increased risk of suicide attempts. Our results showed that the HAMA score was significant higher in suicide attempters than that in non-attempters, and the risk of suicide attempt for patients with high HAMA score was 1.28 times that of patients with low HAMA score. This result is consistent with previous reports showing that anxiety was an important factor for suicide in patients with depression (6). A previous study showed that depressed patients with higher levels of anxiety had more suicide attempts than those without anxiety before and after adjusting for the severity of depression (53). In addition, 79% of patients who committed suicide during hospitalization or immediately after discharge had severe anxiety/agitation symptoms before committing suicide (54). The possible biological mechanisms linking anxiety symptoms to suicide attempts may be explained by both anxiety and suicide associated with serotonergic dysfunction (55). This finding suggests that clinicians should pay more attention to those depressed patients comorbid with anxiety to prevent suicidality, and continue to assess and re-assess anxiety levels to reduce suicidal behavior.

There are several limitations to this study. First, in this study, lipid measurements were only collected at a time-point, but in general, these measurements may change over time. The association between lipid levels and suicide attempts may vary depending on the time points when lipid levels are tested. Moreover, the blood lipid levels of patients with first-onset depression are more likely to be influenced by their diseases. For example, patients do not want to eat and sleep poorly, so lipid disorders occur, leading to the association between lipids and suicide attempts, which is a symptom of depression. Hence, we may have a false lipid-suicide attempt association. We cannot exclude this possibility in this cross-sectional study, and should be further examined in further studies using a longitudinal design. Second, this is a cross-sectional study, with no long-term follow-up cholesterol data. Therefore, the causal relationship between lipid levels and suicidal behavior is still unclear. Third, we collected suicide information through research interviews with patients, family members, and clinicians, rather than using structured tools, which may introduce bias during the collection phase. Fourth, there are too many factors for attempted suicide, but we only included some variables as covariates. Important risk factors for suicide attempts, such as lack of social support, major physical conditions/pain, smoking and drinking, were not controlled in their adjusted analyses (18). Moreover, considering that both nutritional status and stress may affect blood lipid levels, the one time-point measurement of blood lipid levels at admission may not reflect the patient’s baseline level (24). Sixth, there were more female patients in this study, which may be a potential confounder, because the MDD and suicidality are more common in women than men (17). Finally, past suicide attempts may be due to depression or even a depressive episode. Therefore, the diagnosis of the first depressive episode may be questionable in some patients who attempted suicide.

## Conclusion

In summary, our study showed that the prevalence of suicide attempts in FEDN MDD patients was 20.14%. Moreover, high TC and low HDL-c levels were significantly associated with suicide attempts in these patients, suggesting that abnormal lipid levels may be involved in suicide attempt in MDD patients at their early stage. Further, our finding provided further evidence that more severe symptoms of anxiety and depression were associated with suicide attempts in these MDD patients. Therefore, our findings have important implications for clinical care, because the understanding and treatment of depression and anxiety symptoms can provide better results for preventing suicide attempts in FEDN patients with major depression. Due to methodological limitations, longitudinal studies conducted under the control of related variables will more clearly determine the association and causality between abnormal blood lipid levels and suicide attempts in MDD patients in the early stage of the disease. Taken together, understanding the relationship between lipid profiles and suicide attempts in early stage of MDD will provide important implications for the prevention of suicidal behavior.

## Data Availability Statement

The datasets generated for this study are available on request to the corresponding authors.

## Ethics Statement

The studies involving human participants were reviewed and approved by The Institutional Review Board (IRB) of the First Clinical Medical College, Shanxi Medical University. The patients/participants provided their written informed consent to participate in this study.

## Author Contributions

T-QL and X-YZ were responsible for the study design, manuscript preparation and revision. Y-JM and D-FW were responsible for statistical analysis, manuscript preparation and writing the protocol and the paper. Y-JZ, YL, and D-MW were responsible for data collection, lab experiments. All authors contributed to the article and approved the submitted version.

## Funding

This work was supported by the National Key R&D Program of China (2017YFC1310400), and the National Natural Science Foundation of China (81371465 and 81671324). Fundamental Research Founds for the Central South University (1053320183555). This research did not receive any specific grant from funding agencies in the public, commercial, or not-for-profit sectors.

## Conflict of Interest

The authors declare that the research was conducted in the absence of any commercial or financial relationships that could be construed as a potential conflict of interest.

The reviewer, B-LZ, declared a shared affiliation, though no collaboration, with two of the authors, Y-JZ and YL, to the handling Editor.

## Abbreviations

MDD, major depressive disorder; FEDN, first-episode and drug-naïve; OR, odds ratio; BMI, body mass index; HAMD, Hamilton Depression Scale; HAMA, Hamilton Anxiety Scale; HDL, high density lipoprotein; LDL, low density lipoprotein; TC, total cholesterol.
